# Perinatal administration of phencyclidine alters expression of Lingo-1 signaling pathway proteins in the prefrontal cortex of juvenile and adult rats

**DOI:** 10.1042/NS20180059

**Published:** 2018-09-28

**Authors:** Jessica L. Andrews, Kelly A. Newell, Natalie Matosin, Xu-Feng Huang, Francesca Fernandez

**Affiliations:** 1 Centre for Medical and Molecular Bioscience, Faculty of Science, Medicine and Health, University of Wollongong, Wollongong, NSW, Australia; 2 Illawarra Health and Medical Research Institute, University of Wollongong, Wollongong, NSW, Australia; 3 Schizophrenia Research Institute, Sydney, NSW, Australia; 4 School of Psychiatry, Faculty of Medicine, University of New South Wales, Sydney, Australia; 5 Department of Translational Research in Psychiatry, Max Planck Institute of Psychiatry, Munich, Germany; 6 Faculty of Social Sciences, University of Wollongong, Wollongong, NSW, Australia; 7 School of Science, Faculty of Health Sciences, Australian Catholic University, Brisbane, Australia

**Keywords:** Lingo-1 signaling, neurodevelopment, phencyclidine animal model, prefrontal cortex, schizophrenia

## Abstract

Postnatal administration of phencyclidine (PCP) in rodents causes major brain dysfunction leading to severe disturbances in behavior lasting into adulthood. This model is routinely employed to model psychiatric disorders such as schizophrenia, as it reflects schizophrenia-related brain disturbances including increased apoptosis, and disruptions to myelin and plasticity processes. Leucine-rich repeat and Immunoglobin-like domain-containing protein 1 (Lingo-1) is a potent negative regulator of both axonal myelination and neurite extension. The Nogo receptor (NgR)/tumor necrosis factor (TNF) receptor orphan Y (TROY) and/or p75 neurotrophin receptor (p75) complex, with no lysine (K) (WNK1) and myelin transcription factor 1 (Myt1) are co-receptors or cofactors in Lingo-1 signaling pathways in the brain. We have examined the developmental trajectory of these proteins in a neurodevelopmental model of schizophrenia using PCP to determine if Lingo-1 pathways are altered in the prefrontal cortex throughout different stages of life. Sprague–Dawley rats were injected with PCP (10 mg/kg) or saline on postnatal days (PN)7, 9, and 11 and killed at PN12, 5 or 14 weeks for measurement of Lingo-1 signaling proteins in the prefrontal cortex. Myt1 was decreased by PCP at PN12 (*P*=0.045), and at 14 weeks PCP increased Lingo-1 (*P*=0.037), TROY (*P*=0.017), and WNK1 (*P*=0.003) expression. This is the first study reporting an alteration in Lingo-1 signaling proteins in the rat prefrontal cortex both directly after PCP treatment in early development and in adulthood. We propose that Lingo-1 pathways may be negatively regulating myelination and neurite outgrowth following the administration of PCP, and that this may have implications for the cortical dysfunction observed in schizophrenia.

## Introduction

Phencyclidine, also known as PCP, is primarily a potent non-competitive *N*-methyl-d-aspartate (NMDA) receptor antagonist, but is also an agonist for the dopamine D2 receptors [1–3] and to a lesser extent binds opiate, nicotinic, and muscarinic cholinergic receptors [4–7]. Administration of PCP to healthy human subjects induces hallucinations and delusions which are common symptoms of schizophrenia, while PCP administration to schizophrenia patients exacerbates their positive symptoms [8]. Due to its effects on the aforementioned brain receptor targets, which are known to be implicated in the pathology of schizophrenia, PCP treatment in rodents has been used to model the glutamate hypofunction hypotheses and dopamine hyperfunction hypotheses of schizophrenia [2,9]. Since the PCP-induced behaviors in rodents are translatable to the psychomimetic effects in both humans and other higher order primates, the administration of PCP is now one of the best known pharmacological models of schizophrenia [10–12]. The use of PCP at postnatal days (PN)7, 9, and 11 has consistently been shown to induce hyperlocomotion, reduce prepulse inhibition, and impair social interactions in rodents, all of which are behaviors analogous to a number of schizophrenia symptoms observed in humans [13–15].

PCP administration in rodents is reported to cause major disturbances to neuronal cytoarchitecture and plasticity during neurodevelopment across the brain [16,17]. PCP administration at PN7, 9, and 11 has been repeatedly shown to result in an increase in neuronal degeneration in the frontal and cingulate cortex of rats [18,19]. It is hypothesized that disruptions to neuronal structures and neuronal plasticity during this critical perinatal period may be directly responsible for deficits in brain development, and thus may contribute to the appearance of some of the schizophrenia-like symptoms seen in these rats in adulthood. Furthermore, similar to neurons, oligodendrocytes are extremely sensitive to the effects of PCP during development [17], and myelination has been shown to be significantly affected by PCP treatment both *in utero* and postnatally [16,17]. We have previously shown in this rat model that myelin basic protein (MBP), a marker of mature oligodendrocytes and myelination, is significantly reduced in early development by perinatal administration of PCP [20]. Considering the significant role of oligodendrocytes in axonal connectivity, conduction and myelination, disruption to these critical processes during early neurodevelopment can have significant negative consequences, affecting normal brain development.

Leucine-rich repeat and Ig domain-containing protein (Lingo-1) pathways are responsible for regulating levels of myelination and neuronal growth in the brain, which are processes impaired in schizophrenia. Lingo-1 is expressed on both neurons and oligodendrocytes [21]; it acts through a trimolecular complex both with the Nogo receptor (NgR) co-receptor and either the p75 neurotrophin receptor (p75) or its functional homolog, tumor necrosis factor (TNF) receptor orphan Y (TROY) [22–24]. Together this signaling complex activates ras homolog gene family, member A (RhoA) leading to the inhibition of both neuronal growth and myelination related processes [21,25]. Lingo-1 signaling through additional cofactors such as with no lysine (K) (WNK1), myelin transcription factor 1 (Myt1) and its homolog Myt1-like (Myt1l) also lead to the regulation of myelination and neurite outgrowth [26–28].

Studies in a healthy adult postmortem human brain have shown that expression of cortical Lingo-1 transcripts are amongst the highest in the brain [29]. Considering the high degree of identity between human and mouse *Lingo-1* orthologs (99.5%), and that *Lingo-1* transcript levels were reported to be highly expressed in the cortical regions of both rat and mouse brains in adulthood and throughout neurodevelopment [29,30], the perinatal PCP neurodevelopmental model seems ideal for studying the developmental trajectory of Lingo-1 expression in the context of schizophrenia.

We have recently provided the first evidence of an alteration in Lingo-1 signaling pathways in the postmortem dorsolateral prefrontal cortex (DLPFC) in schizophrenia [31]. Bearing in mind the role of Lingo-1 signaling proteins in myelin-related processes, and the fact that we found Lingo-1 protein expression to be significantly up-regulated in the human DLPFC in schizophrenia [31], the present study specifically focusses on Lingo-1 signaling protein alterations in the prefrontal cortex of the rats in our model. Considering that the perinatal administration of PCP to rodents is a well-established developmental animal model for schizophrenia, we sought to investigate the effects of perinatal PCP administration on levels of expression of Lingo-1 signaling proteins in the prefrontal cortex, a critical region for cognitive processing that is consistently reported to be disrupted in schizophrenia.

## Experimental

### Ethical statement

The present study was approved by the Animal Ethics Committee at The University of Wollongong (AE13/01), and was conducted according to the guidelines of the Australian code of Practice for the Care and Use of Animals for Scientific Purposes, 8th edition (2013), conforming to the International Guiding Principles for Biomedical Research Involving Animals. All efforts were made to minimize numbers of animals used and their suffering.

### Animals

**Figure 1:**
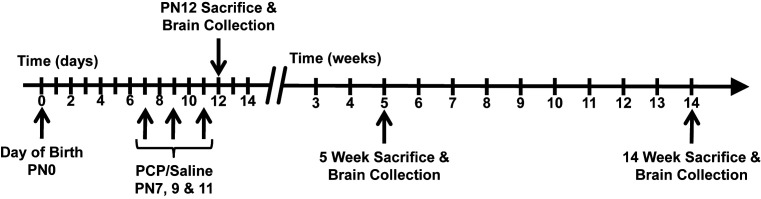
Timeline of experimental procedures Day of birth was denoted as PN0. Male rat pups received subcutaneous injections of PCP (10 mg/kg) or saline (0.9%, 1 ml/kg) at PN7, 9, and 11. Rats were killed and brains were collected for processing at PN12, 5 and 14 weeks of age, representing juvenile, adolescent, and adult stages of life, respectively.

Timed pregnant Sprague–Dawley rats were obtained at gestation day 14 from the Animal Resource Centre (Perth, WA, Australia). Rats were housed in environmentally controlled conditions at 22°C in a 12:12-h light/dark cycle with food and water access *ad libitum*. The day of birth was denoted as PN0 (Figure 1), and the pups were sexed on PN7 when the litters were subsequently randomly assigned to PCP or saline groups. The female pups remained in the litters until weaning, however only male rats were used in the present study. The pups were weaned at PN24–28, and were housed in pairs according to treatment.

### Experimental design

The pups received a single daily subcutaneous injection, administered between 08:00 and 09:00 h, on PN7, 9, and 11; of PCP (10 mg/kg/day; Sigma, Castle Hill, NSW, Australia) or saline (0.9% NaCl at a volume of 1 ml/kg) (Figure 1). The acute effects of PCP administration were validated by observing an immediate increase in locomotor activity and a lack of huddling for the few hours immediately following injections in the PCP treated pups compared with saline treated pups, as previously described [32]. Six rats from each treatment group (PCP and control) were killed by decapitation at PN12 days or by CO_2_ asphyxiation at 5 and 14 weeks of age, representing perinatal, adolescent, and adult developmental stages, respectively, as described previously [33–35] (Figure 1). These time points were chosen specifically because they are important and distinct developmental periods in life [36] and are particularly relevant to the pathophysiology of schizophrenia. Brains were extracted and the prefrontal cortex was regionally dissected on ice with the aid of a standard rat brain atlas [37]. Immediately following dissection, samples were snap-frozen in liquid nitrogen and then stored at −80°C until use. The prefrontal cortex was specifically examined in the present study for two reasons; first it is a region highly implicated in the schizophrenia pathophysiology [38], and second we have previously shown Lingo-1 and its signaling partners to be significantly altered in the prefrontal cortex of postmortem schizophrenia brains compared with controls [31].

Tissue was gently homogenized in lysis buffer (50 mM Tris pH 7.5, 50% glycerol), containing aprotinin and a protease inhibitor cocktail (Sigma). Protein concentrations were determined by a spectrophotometer and all samples were subsequently diluted to a concentration of 2 μg/μl. A total of 10 µg protein from each sample was separated by electrophoresis on 4–12% pre-cast Bis-Tris polyacrylamide gels (Bio-Rad). All samples were run in duplicate or triplicate across four to six gels per protein, and were loaded in a randomized order with even numbers of PCP and control samples per time point per gel to minimize the effects of gel-to-gel variability on the results. A pooled sample was used as a positive control and was loaded on to each gel within the experiment to account for any gel-to-gel variability which was calculated to be between 1.1 and 14.8%. Proteins were subsequently transferred to PVDF membranes (Bio-Rad). The resultant membranes were blocked using 5% BSA for 1 h, followed by overnight incubation at 4°C with primary antibodies in 1% BSA for each of the proteins of interest. Following 3 × 5 min washes in PBS + 0.1% Tween 20 (PBST) membranes were incubated with horseradish conjugated secondary antibodies for 1 h at room temperature. The Gel Logic 2200 Pro (Carestream Molecular Imaging; Rochester, NY, U.S.A.) was used to visualize and quantitate the bands of interest. Samples from each gel were then normalized to their respective pooled sample, and all bands were normalized to same lane β-actin loading control. Mean β-actin expression levels did not differ between PCP and control groups (*P*>0.05), however mean β-actin expression levels were found to be affected by age (*P*<0.05). All experiments and quantitations were performed blind to treatment and age group.

### Antibodies

The polycolonal antibodies for Lingo-1 (ab23631), NgR (ab26291), p75 (ab8874), TROY (ab12126), and Myt1 (ab82844), and monoclonal antibody for WNK1 (ab128858) were all purchased from Abcam (Melbourne VIC, Australia). Primary antibody dilution ranged from 1:200 to 1:500. Secondary antibodies for rabbit (AP307P) and mouse (AP308P) were purchased from Merck Millipore (Bayswater, VIC, Australia) and were used at a concentration of 1:3000. The monoclonal antibody for β-actin (MAB1501) was also purchased from Merck Millipore and was used at a concentration of 1:5000. Antibody specificity has been previously demonstrated by the use of appropriate positive controls as documented in both the literature [33,39–44] and the antibody datasheets provided by Abcam.

### Statistics

Statistical analyses were performed using SPSS (version 20.0, SPSS Inc. Chicago, U.S.A.). As all data were normally distributed (Kolmogorov–Smirnov 0.255≤*P*≤0.891), parametric testing was implemented. Two-way multivariate ANOVAs (MANOVA) followed by Tukey’s HSD post-hoc tests, were performed to assess interactions between treatment (PCP or control) and time point (PN12, 5 or 14 weeks). One-way ANOVA followed by Tukey’s HSD post-hoc tests were used to assess for differences in protein expression across successive developmental time points within each treatment group; however since β-actin was found to be altered across developmental time points, the effects of age on protein expression must be considered with caution. Unpaired two-tailed *t*tests were also used at individual time points to assess differences in protein expression between PCP and control groups. Since we have previously reported expression levels of the myelin related proteins MBP and myelin oligodendrocyte glycoprotein (MOG) in this cohort of rats [20], we performed Pearson’s correlations to examine the relationship between the expression of Lingo-1 and these myelination related proteins across developmental time points in both control and PCP treated groups of rats. The significance for all statistical tests was set to *P*<0.05. All data were expressed as mean ± S.D.

## Results

### Protein detection

**Figure 2:**
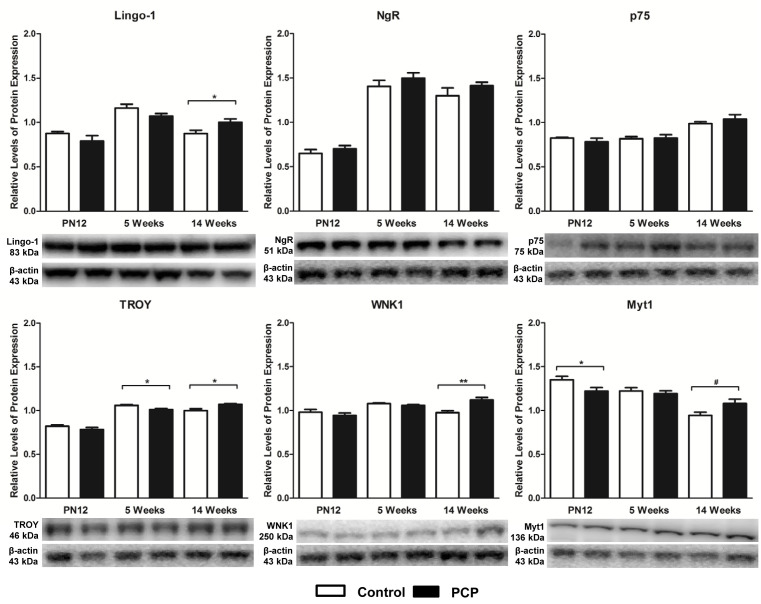
Relative levels of expression of Lingo-1 signaling proteins in the prefrontal cortex of rats at juvenile (PN12), adolescent (5 weeks), and adult (14 weeks) stages of life following perinatal administration of PCP (10 mg/kg) or saline (control; 1 ml/kg, 0.9% NaCl) at PN 7, 9, and 11 Graphs depict levels of protein expression normalized to β-actin same lane loading controls from control (white bars) and PCP treated rats (black bars). Representative immunoblot bands for all proteins of interest and β-actin bands are depicted below; *n*=6 rats per treatment per time point. ^#^Statistical trend; *P<0.05 and **P<0.01.

Lingo-1 and NgR were each detected as a single specific band at 83 and 51 kDa, respectively as has been previously reported [33,42,43] (Figure 2). In addition to the specific bands for p75 (75 kDa), TROY (46 kDa), WNK1 (250 kDa), and Myt1 (135 kDa), a number of non-specific bands were also observed with the use of these antibodies. As mentioned above, the specificity of these antibodies has been previously demonstrated by the use of appropriate positive controls in the literature [33,39–44] and the suppliers’ antibody datasheets; therefore the single bands corresponding to the expected molecular weights were the bands quantitated in the present study (Figure 2).

### Levels of Lingo-1 and signaling partners TROY, WNK1, and Myt1 protein expression are altered in the prefrontal cortex of rats throughout development by perinatal administration of PCP

**Figure 3:**
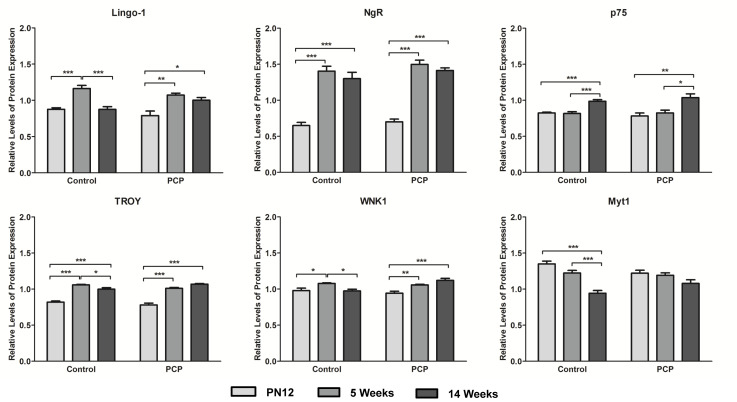
Developmental expression profile of Lingo-1, NgR, p75, TROY, WNK1, and Myt1 proteins in the prefrontal cortex of control (saline) and PCP treated rats at PN12 (light gray), 5 week (medium gray), and 14 week (dark gray) time points (*n*=6 rats per time point) *P<0.05, **P<0.01, and ***P<0.001.

There was a significant age–treatment interaction on levels of Lingo-1 protein expression (F_2,30_ = 4.701; *P*=0.017). Post-hoc analyses revealed that this interaction occurs at 14 weeks of age, and that Lingo-1 levels are significantly increased by 14.5% in PCP treated rats compared with controls (*P*=0.037; Figure 2). While there was no significant main effect of treatment on protein expression (F_1,30_ = 0.255; *P*=0.617), there was a highly significant main effect of age on levels of Lingo-1 protein expression (F_2,30_ = 25.247; *P*<0.001). Within the control group, there was a significant 33% increase in Lingo-1 expression in the 5-week-old rats compared with the PN12 rats (*P*<0.001; Figure 3), and a 24.5% decrease in Lingo-1 expression in the 14-week rats compared with the 5-week rats (*P*<0.001; Figure 3). Furthermore, in the PCP treated group, there was a significant 36% increase in Lingo-1 expression in the 5 week group compared with PN12 (*P*=0.001; Figure 3), and a 20% increase in the 14 week group compared with PN12 (*P*=0.012; Figure 3).

There was also a significant age × treatment effect on the protein expression levels of the Lingo-1 signaling partners TROY (F_2,30_ = 8.274; *P*=0.001), WNK1 (F_2,30_ = 9.094; *P*=0.001), and Myt1 (F_2,30_ = 5.594; *P*=0.009). In all three cases, there was an increase in protein expression in the adult PCP treated rats compared with the control group, despite the effect on TROY protein expression only being a minor increase of 7% (*P*=0.011; Figure 2), and the effect on Myt1 protein expression only equating to a borderline significant increase of 14.5% (*P*=0.056; Figure 2). The most significant age–treatment interaction on the three signaling partners was the 15% increase in WNK1 protein expression in the 14-week-old PCP treated rats compared with controls (*P*=0.003; Figure 2). There was also a very minor but significant 4.5% decrease in TROY expression in PCP treated rats compared with controls at 5 weeks of age (*P*=0.011; Figure 2), and a significant 9.5% decrease in Myt1 expression in PN12 PCP treated rats compared with controls (*P*=0.045; Figure 2). As with Lingo-1, there was no main effect of treatment on protein expression for TROY (F_1,30_ = 0.183; *P*=0.672), WNK1 (F_1,30_ = 2.417; *P*=0.130), or Myt1 (F_1,30_ = 0.063; *P*=0.803); however in all three cases, there was a highly significant main effect of age on protein expression (TROY: F_2,30_ = 138.129; *P*<0.001, WNK1: F_2,30_ = 11.254; *P*<0.001, and Myt1: F_2,30_ = 25.047; *P*<0.001). Post-hoc analyses revealed that for TROY, within both the control group and PCP treated group, these changes resulted in an increase in TROY expression in the 5-week group compared with the PN12 group (control: +29%; *P*<0.001, PCP: +29.5%; *P*<0.001; Figure 3), and an increase in TROY expression in the 14-week group compared with the PN12 group (control: +22%; *P*<0.001, PCP: +37%; *P*<0.001; Figure 3). Additionally, there was a small but significant decrease in TROY expression in the 14-week control rats compared with the 5-week control rats (–5.5%; *P*=0.038; Figure 3). With regard to WNK1 expression, there was a significant increase in the 5-week group compared with PN12 (control: +10%; *P*=0.031, PCP: +12%; *P*=0.009; Figure 3). There was also a significant decrease in WNK1 expression in the 14-week control rats compared with the 5-week control rats (–9.5%; *P*=0.022; Figure 3), and a significant increase in WNK1 expression in the 14-week PCP treated rats compared with the PN12 PCP treated rats (+19%; *P*<0.001; Figure 3). Last, Myt1 expression levels were only found to be affected by age in the control rats. There was a highly significant decrease in Myt1 expression in the 14-week rats compared with the PN12 rats (–30%; *P*<0.001; Figure 3) and in the 14-week rats compared with the 5-week rats (–23%; *P*<0.001; Figure 3). There was no significant difference in Myt1 protein expression observed across any of the tested age groups within the PCP treated rats (0.074≥*P*≥0.878).

### Cortical levels of NgR and p75 proteins are unaltered by perinatal PCP treatment

There were no significant interactions between age and treatment on levels of NgR or p75 (F_2,30_ = 0.147; *P*=0.864 and F_2,30_ = 0.928; *P*=0.406, respectively). Furthermore there were no main effects of treatment on levels of NgR (F_1,30_ = 3.242; *P*=0.082; Figure 2) or p75 (F_1,30_ = 0.034; *P*=0.855; Figure 2) protein expression in the prefrontal cortex of the treated rats. However, an extremely significant age effect was observed in relation to both NgR (F_2,30_ = 104.653; *P*<0.001) and p75 (F_2,30_ = 22.782; *P*<0.001) protein levels in the prefrontal cortex of the treated rats. Post-hoc analyses demonstrated that this age effect resulted in an immense increase in NgR expression levels in both control and PCP treated rats in both the 5-week old rats compared with the PN12 rats (control: +116%; *P*<0.001, PCP: +113.5%; *P*<0.001; Figure 3), and in the 14-week old rats compared with the PN12 rats (control: +100%; *P*<0.001, PCP: +101.5%; *P*<0.001; Figure 3). Alterations in p75 were still highly significant, although the magnitude of change was less than that of NgR. In both the control and PCP groups, a significant increase in p75 protein expression was observed in the 14-week age group compared with the PN12 age group (control: +19.5%; *P*<0.001, PCP: +32.5%; *P*=0.003; Figure 3), as well as in the 14-week age group compared with the 5 week age group (control: 21%; *P*<0.001, PCP: +25.5%; *P*=0.010; Figure 3).

### The relationship between Lingo-1 protein expression and myelination related proteins MBP and MOG throughout neurodevelopment in control and PCP treated rats

Pearson’s correlations revealed that there were no significant relationships between levels of Lingo-1 protein expression and either of the myelin related proteins MBP or MOG in either control or PCP treated rats at PN12 and 5 weeks of age (Table 1). Furthermore, there was no significant correlation between Lingo-1 and MBP levels in either the 14-week-old control or PCP treated rats (Table 1). However there was a strong significant negative correlation between Lingo-1 and MOG expression levels in the PCP treated rats at 14 weeks of age (Table 1).

**Table 1:** Pearson’s correlations for associations between levels of expression of Lingo-1 and myelin and oligodendrocyte related proteins – MBP and MOG, in the prefrontal cortex of perinatal PCP (10 mg/kg) and saline treated rats at PN12, 5 and 14 weeks

	PN12		5 weeks		14 weeks	
	Control	PCP	Control	PCP	Control	PCP
MBP	r = 0.763	r = 0.728	r = 0.464	r = 0.656	r = 0.108	r = −0.728
	*P*=0.078	*P*=0.101	*P*=0.355	*P*=0.157	*P*=0.838	*P*=0.102
MOG	r = 0.311	r = 0.474	r = 0.652	r = 0.060	r = 0.568	**r = −0.885**
	*P*=0.548	*P*=0.343	*P*=0.161	*P*=0.911	*P*=0.240	***P*=0.019**

There was a significant negative correlation between Lingo-1 and MOG protein expression in PCP treated rats at 14 weeks (r = −0.885, *P*=0.019; highlighted in bold).

## Discussion

Postnatal administration of PCP in rodents causes major disturbances to neurobiological processes including neurite outgrowth and myelination, both of which are integral to the neurodevelopmental hypothesis for schizophrenia [45–47]. Here we provide the first report of developmental alterations of the expression of Lingo-1 signaling proteins in the prefrontal cortex in a validated neurodevelopmental animal model of schizophrenia. We have shown that levels of Lingo-1, TROY, and WNK1 proteins were significantly increased in the prefrontal cortex of rats treated perinatally with PCP in adulthood compared with their controls. Additionally, Myt1 was significantly decreased in juvenile PCP treated rats at PN12 compared with control rats. Despite these significant alterations to Lingo-1 signaling proteins, the co-receptors NgR and p75 were not found to be significantly altered by PCP treatment across any of the three tested developmental time points. We have recently provided the first evidence of alterations of Lingo-1 signaling pathway proteins in the postmortem schizophrenia prefrontal cortex [31]. Considering current literature, the results of the present study not only validate the PCP rodent model for use in the study of Lingo-1 signaling proteins in the context of schizophrenia, but also suggest that the PCP rodent model is a suitable preclinical model to assess novel therapeutic agents targetting Lingo-1 signaling to potentially combat the dysregulation of myelination and neurite outgrowth seen in schizophrenia.

### Perinatal PCP administration affects cortical expression of Lingo-1 signaling proteins at juvenile and adult stages of life

Perinatal PCP treatment was found to significantly increase levels of Lingo-1 protein expression in adult rats, a result that is in line with our previous finding of a significant increase in Lingo-1 protein expression in the DLPFC of postmortem schizophrenia brains compared with controls [31]; however Lingo-1 expression was not significantly altered in the earlier stages of rodent neurodevelopment by perinatal PCP administration. While no immediate effect of PCP treatment was reported for Lingo-1 expression at PN12, Myt1 protein expression was found to be significantly decreased in the prefrontal cortex of PCP treated rats at PN12. Since Myt1 is known to play an essential role in the maturation of oligodendrocytes during development [48], this result is also in parallel with our previous reports of a decrease in the expression of MBP, a marker of mature oligodendrocytes and myelination, in the same region using the same drug administration paradigm [20].

Despite having previously reported an alteration of the mature oligodendrocyte and myelination marker MBP in the prefrontal cortex of juvenile PCP treated rats [20], it appears that the blockade of NMDARs by PCP early in neurodevelopment may only result in the dysregulation of Lingo-1 later in life, and thus may only affect the regulation of Lingo-1 myelin related processes in adulthood. This is supported by a significant negative correlation between Lingo-1 and MOG expression levels in the PCP treated 14-week-old rats reported in the present study, while no significant correlations were observed between Lingo-1 and either MBP or MOG at any other stage of development in control or PCP treated rats. It is possible that PCP-induced Lingo-1 related disturbances early in life are masked by dysregulation of other myelin regulating proteins, such as the receptor tyrosine-protein kinase ErbB4, a well-established receptor involved in positively regulating myelination, notably during neurodevelopment. Previous studies by our research group using the same PCP animal model as that of the present study, have reported a significant decrease in levels of ErbB4 expression in the prefrontal cortex of PCP treated rats at PN12 (−25%; *P*<0.05), which persisted, although slightly attenuated, into adulthood (−18%; *P*<0.01) [34]. These results in conjunction with those of the present study demonstrate that while ErbB4 was strongly altered by PCP treatment immediately after treatment and was sustained into adulthood, Lingo-1 dysfunction by PCP administration was only detectable later in life. Due to the high levels of plasticity occurring in this brain region in the early stages of neurodevelopment, it is possible that compensatory mechanisms may have masked alterations in Lingo-1 expression induced by PCP in the juvenile rats. However, in the later stages of life when neuron development is complete, Lingo-1 and a number of its co-receptors/co-signaling partners were significantly affected, as demonstrated by the results of the present study. In addition, the developmental expression profile of Lingo-1 protein throughout healthy brain development, as demonstrated in our control rats, showed an increase in Lingo-1 expression at 5 weeks compared with PN12, with Lingo-1 levels decreasing in adulthood and returning to levels close to those of juvenile rats (Figure 3). Similarly, in the PCP treated rats the levels of Lingo-1 were also elevated at 5 weeks compared with PN12, although to a lesser extent; however in contrast with the control rats, Lingo-1 levels remained high into adulthood. These results support our hypothesis that Lingo-1 pathway dysregulation is only detectable at the adult stage of life; however since the β-actin loading control was found to be significantly affected by age in the present study, these results must be considered with caution.

Levels of TROY expression were also found to be significantly increased in adult PCP treated rats compared with controls in the present study. TROY is widely expressed in adult neurons where it can substitute for p75 in the Lingo-1/NgR/p75 signaling complex in the presence of myelin associated inhibitors in p75 deficient neurons [24,49–51]. Since p75 levels were found to be unaltered in the adult rats by perinatal PCP treatment in the present study, we would suspect that in this instance, TROY has substituted for p75 within the Lingo-1/NgR/p75 receptor complex on neurons in the adult PCP treated rats. Considering TROY and Lingo-1 are expressed together in certain subpopulations of neurons, in addition to reactive astrocytes, and macrophages/microglia, but not in oligodendrocytes [52,53], it could be hypothesized that the concurrent up-regulation of these two proteins in the prefrontal cortex in adult rats following perinatal PCP administration are playing a role in negatively regulating neurite outgrowth in the prefrontal cortex of our adult PCP treated rats. In support of this, dominant-negative forms of TROY, in addition to *TROY* knockout mice, have reduced levels of activated RhoA and enhanced neurite outgrowth in the presence of myelin inhibitors [24,49,54].

Lingo-1 has also been shown to directly bind to epidermal growth factor receptor (EGFR), negatively regulating the EGFR/PI3-K/protein kinase B (PKB) (Akt) signaling pathway, where it has been found to regulate dopamine neuron survival, growth, and function [55]. It was found that all of these factors were improved following Lingo-1 antagonism, and that inhibiting Lingo-1 also increased both EGFR and p-Akt levels in the absence of myelin inhibitors promoting retinal cell survival [55,56]. We hypothesize that the increased levels of Lingo-1 protein expression in the prefrontal cortex of PCP treated rats may be negatively regulating the EGFR/phosphatidylinositide 3-kinase (PI3K)/Akt signaling pathway, whereby it would impede neuronal growth and survival. This hypothesis is supported by previous findings from our research group showing that phosphorylated levels of Akt were significantly decreased in the prefrontal cortex of adult PCP treated rats using the same treatment regime [34]. In the present study, Myt1 was found to be increased in the prefrontal cortex of adult PCP treated rats by the same magnitude as that of Lingo-1, despite not reaching statistical significance (*P*=0.056). This is in line with our finding of a concurrent increase in both Lingo-1 and Myt1 protein expression in the postmortem DLPFC of schizophrenia patients compared with controls [31], and is further supported by a study that has identified microduplications in the *Myt1l* gene in 2% of childhood onset schizophrenia subjects from the largest cohort of very-early onset childhood onset schizophrenia subjects to date [57]. Additionally, both *Lingo-1* and *Myt1* gene expression have been found to be increased when adult nerve cells were exposed to traumatic injuries, and in demyelinated lesions in rodent and human central nervous system injuries, respectively [30,58], indicating that the concurrent up-regulation of Lingo-1 (and potentially Myt1) observed in the present study may be playing a significant role in regulating neuronal survival and myelination processes in the prefrontal cortex of our PCP treated rats.

The final Lingo-1 signaling protein to be significantly altered by PCP administration in the present study was WNK1, a Lingo-1 binding partner shown to be co-localized with Lingo-1 in cortical cultured neurons [28]. WNK1 expression was found to be significantly increased in the prefrontal cortex of the adult PCP treated rats compared with control rats in our neurodevelopmental rat model. In support of this finding, *WNK1* gene expression has been consistently reported to be up-regulated in the prefrontal cortex of schizophrenia sufferers in genome-wide expression profiling studies [59,60], thus providing support for a dysregulation of WNK1 in schizophrenia.

Cortical levels of the Lingo-1 co-receptors NgR and p75 were not significantly altered by PCP treatment at any of the three developmental time points (Figure 2), this is in accordance with two previous studies showing that *NgR* mRNA expression is not altered following PCP treatment in rats [61], and that p75 protein expression is not altered by PCP treatment in cortical cultured neurons [62].

### Limitations

Owing to the limited quantities of prefrontal cortex tissue obtained from these rats, it was not possible to perform any histological testing to examine the cellular locations (i.e. neurons compared with oligodendrocytes) of Lingo-1 or its signaling partners to support our proposed hypotheses regarding the effect of Lingo-1 depending on its cellular location, or to examine the structural integrity of the myelin sheaths in this model (e.g. using TEM). Further studies will be necessary to better characterize these protein–protein interactions under the influence of PCP at a molecular level. Furthermore, since the β-actin loading control was found to be significantly affected by age in the present study, the results pertaining to protein expression alterations across developmental time points must be considered with caution.

## Conclusion

In summary, we report for the first time alterations in Lingo-1 signaling pathway proteins in the prefrontal cortex of rats from a neurodevelopmental PCP model of schizophrenia. We have shown an altered developmental trajectory of Lingo-1 and a number of its signaling partner proteins after PCP treatment, predominantly during the adult stage of life. Our results in concert with current literature validate the PCP rodent model for use in the study of Lingo-1 signaling proteins in the context of schizophrenia. Furthermore, due to the role of the many Lingo-1 pathways as a negative regulator of myelination and neurite outgrowth, and considering the implication of both of these central processes in cognitive performance, antagonists of Lingo-1 may be potential candidates for novel therapeutic agents for the treatment of the cognitive dysfunction in schizophrenia. The present study demonstrates that the PCP rodent model is a suitable preclinical model to assess such novel therapeutic agents targeting Lingo-1 signaling pathways to potentially combat the dysregulation of myelination and neurite outgrowth resulting in the cognitive deficits seen in schizophrenia.

## Summary

Myelination (the addition of a protective insulating layer to neurons) and neuronal outgrowth are both processes occurring during brain development and previously implicated in schizophrenia. As schizophrenia can originate from changes occurring during the embryonic development, it is vital to study genes and proteins involved in the development of the nervous system as their dysfunction may be an underlying cause of the disorder. The broad function of Lingo-1 and its partner proteins is to regulate key brain mechanisms underlying myelination and neuronal outgrowth. We have used a rat model mimicking dysfunction at the embryonic level and resulting in schizophrenia pathology later in life. We have studied the levels of expression of these proteins throughout critical periods of development, specifically the postnatal, adolescent, and adult stages of life in this rat model. We have shown that Lingo-1 and its partner proteins are significantly altered throughout development in this rodent model of schizophrenia in a brain region critical to cognitive function, suggesting that Lingo-1 pathway may have implications for the developmental processes associated with cognitive dysfunction observed in schizophrenia.

## Abbreviations

Akt: protein kinase B (PKB)
DLPFC: dorsolateral prefrontal cortex
EGFR: epidermal growth factor receptor
Lingo-1: leucine-rich repeat and Ig domain-containing protein
MBP: myelin basic protein
MEK1: mitogen activated protein kinase-1
MOG: myelin oligodendrocyte glycoprotein
Myt1: myelin transcription factor 1
Myt1l: Myt1-like
NgR: Nogo receptor
NMDA: *N*-methyl-d-aspartate
NMDAR: NMDA receptor
p75: p75 neurotrophin receptor
PCP: phencyclidine
PN: postnatal day
RhoA: ras homolog gene family, member A
TROY: tumor necrosis factor receptor super family, member 19
WNK1: with no lysine (K)
